# Patient Perspectives of Bowel Urgency and Bowel Urgency-Related Accidents in Ulcerative Colitis and Crohn’s Disease

**DOI:** 10.1093/ibd/izae044

**Published:** 2024-03-21

**Authors:** Vipul Jairath, Theresa Hunter Gibble, Alison Potts Bleakman, Kaitlin Chatterton, Paolo Medrano, Megan McLafferty, Brittany Klooster, Sonal Saxena, Richard Moses

**Affiliations:** Division of Gastroenterology, Department of Medicine, Western University, London, Ontario, OX3 9DU, Canada; Eli Lilly and Company, Indianapolis, IN, USA; Eli Lilly and Company, Indianapolis, IN, USA; Patient-Centered Outcomes, Adelphi Values, Boston, Massachusetts, USA; Patient-Centered Outcomes, Adelphi Values, Boston, Massachusetts, USA; Patient-Centered Outcomes, Adelphi Values, Boston, Massachusetts, USA; Patient-Centered Outcomes, Adelphi Values, Boston, Massachusetts, USA; Eli Lilly and Company, Indianapolis, IN, USA; Eli Lilly and Company, Indianapolis, IN, USA

**Keywords:** ulcerative colitis, Crohn’s disease, bowel urgency, patient-reported outcomes

## Abstract

**Background:**

Bowel urgency is bothersome in patients with ulcerative colitis (UC) or Crohn’s disease (CD) and impacts their well-being but remains underappreciated in clinical trials and during patient–healthcare provider interactions. This study explored the experiences of bowel urgency and bowel urgency-related accidents to identify the concepts most relevant and important to patients.

**Methods:**

Adults with a diagnosis of moderate-to-severe UC or CD for ≥6 months and experience of bowel urgency in the past 6 months were included. Qualitative, semi-structured interviews were conducted via telephonic/Web-enabled teleconference. Interview transcripts were coded and analyzed in ATLAS.ti 9 using a systematic thematic analysis.

**Results:**

In total, 30 participants with UC or CD (n = 15 each) (mean age 52 and 50 years, respectively) participated in the interviews. The majority of participants were receiving biologic and/or conventional therapy (80% and 87%, respectively). Most participants with UC (87%) and all with CD experienced bowel urgency-related accidents. The most frequently reported symptoms co-occurring with bowel urgency were abdominal pain, fatigue, and abdominal cramping. Abdominal pain and abdominal cramping were the most bothersome co-occurring symptoms of bowel urgency and bowel urgency-related accidents. In both groups, participants reported decreased frequency of bowel urgency and not wanting to experience bowel urgency-related accidents at all as a meaningful improvement.

**Conclusions:**

Participants with UC or CD expressed bowel urgency and bowel urgency-related accidents to be bothersome and impactful on their daily lives despite use of biologic and/or conventional therapy. These findings underscore the need for development of patient-reported outcome measures to assess bowel urgency in clinical settings.

**Key Points**

- Bowel urgency, the sudden and immediate need to have a bowel movement, and bowel urgency-related accidents are bothersome and impactful symptoms of ulcerative colitis and Crohn’s disease but are underrecognized in patient–healthcare provider interactions and clinical trials.
- This study indicates that bowel urgency and bowel urgency-related accidents are accompanied by several symptoms (abdominal pain, diarrhea, etc.) and affect multiple aspects of patients’ daily lives (work, emotional functioning, social activities, sexual health, etc.).
- These results highlight significant disease burden due to bowel urgency and bowel urgency-related accidents, underscoring the need for evaluation and response to intervention of bowel urgency in clinical settings.

## Introduction

Inflammatory bowel disease (IBD) comprises a group of chronic idiopathic disorders characterized by intestinal inflammation.^1,2^ The most common forms of IBD are ulcerative colitis (UC) and Crohn’s disease (CD).^3^ IBD symptoms include increased stool frequency, diarrhea, abdominal discomfort, rectal bleeding, reduced appetite, fatigue, bowel urgency, and fecal incontinence.^4,5^ Among these, bowel urgency (also known as rectal urgency or fecal urgency), the sudden and immediate need to have a bowel movement, is bothersome for patients often due to the fear of fecal incontinence.^6-8^

More than 80% of patients with UC^8-11^ and 60% to 74% of patients with CD^9-14^ experience bowel urgency, with 25% to 50% of patients experiencing bowel urgency at least once daily. Bowel urgency contributes to a considerable amount of disease-related stress resulting from various situations involving episodes of fecal incontinence, fear of using public toilets, and adaptive behaviors, such as bathroom mapping (knowing the location of the nearest bathroom), wearing an adult diaper, or carrying a cleanup kit.^15-20^ Such psychological stress associated with the experience or fear of bowel urgency-related accidents severely impacts patients’ well-being, which in turn can aggravate IBD symptoms.^21-23^

Despite the significant impact of bowel urgency on health-related quality-of-life (HRQoL) in patients with IBD, it has not been addressed effectively, and its assessment was previously not a recommended endpoint in clinical trials.^24,25^ Given the importance of bowel urgency for patients, the recently updated Food and Drug Administration guidelines for UC and CD encourage the exploration of bowel urgency.^24,25^ Further, bowel urgency is often ignored during patient–healthcare provider interactions due to the embarrassment associated with it; therefore, patients’ individual concerns may remain unaddressed.^26,27^ In this context, patient-reported outcome (PRO) measures are valuable tools for quantifying the patient perspectives of bowel urgency.

Bowel urgency is associated with decreased HRQoL in patients with UC or CD and future risk of hospitalizations, use of corticosteroids, and colectomy for patients with UC.^28,29^ Therefore, it must be considered as a UC- or CD-specific PRO to assess disease severity and should be included in clinical trials. In accordance with Food and Drug Administration’s guidance to include PRO measures in drug development,^30^ measures such as the Urgency Numeric Rating Scale (NRS), Crohn’s Disease Diary, Symptoms and Impacts Questionnaire–UC, Symptoms and Impacts Questionnaire–CD, CD‐PRO/Signs and Symptoms diary, UC‐PRO/Signs and Symptoms diary, PRO-UC diary, and PRO-CD diary have recently been developed.^11,16,19,31-35^ Although these measures include bowel urgency, other IBD symptoms are also included in these tools, except in the Urgency NRS, the only single-item PRO measure assessing bowel urgency severity that is psychometrically validated for UC and content-validated for CD.^16,36^ On the contrary, while other PRO instruments include fecal incontinence/accidents as an endpoint, the Urgency NRS does not.

Understanding patients’ perspectives and identifying meaningful and relevant concepts is of utmost importance for using PROs as clinical trial endpoints during drug development. The present study explored the patient experiences of bowel urgency and bowel urgency-related accidents by gathering descriptive information from patients with UC or CD. The findings of this study will provide insights for the development of a UC- or CD-specific PRO measure to assess severity and meaningful improvement in bowel urgency and bowel urgency-related accidents and help clinicians to discuss and determine appropriate treatment regimens.

## Methods

A brief overview of the study methodology is presented in Supplementary Figure 1.

### Study Population

Recruitment of study participants was conducted by Adelphi Values. The study included adult participants (≥18 years of age) who were diagnosed with moderate-to-severe UC or CD for ≥6 months; had experienced bowel urgency in the past 6 months; were able to read, write, and fluently speak in U.S. English; and were willing and able to participate in a 90-minute telephone interview.

For both UC and CD, the clinician reported the disease severity of participants using the Physician Global Assessment. For participants with CD, the clinician selected a description that best described the patient’s phenotype. Clinicians also recorded if the participants had experienced bowel urgency in the past 3 months. Participants were excluded if they had undergone surgery as part of treatment of UC or CD (eg, ileostomy, colostomy, intra-abdominal surgery) or had a condition or situation (eg, cognitive impairment) that posed a significant risk, confounded the study results, or interfered significantly with their participation in the study. The eligibility of each participant was confirmed by Adelphi Values by verifying the Inclusion and Exclusion Criteria Document filled by the clinician and the Demographic and Health Information Form and informed consent forms. All participants were provided with a nominal honorarium after completion of the interview.

### Ethical Considerations

All study documents were reviewed by a centralized independent review board, Sterling Independent Review Board (https://sterlingirb.com/). All interviews were anonymized to preserve participants’ confidentiality. Patients provided their consent to participate in the interviews in accordance with the Health Insurance Portability and Accountability Act.^37^

### Concept Elicitation Interviews

Concept elicitation interviews (CEIs) were conducted via telephone or Web-enabled teleconference from September 27, 2021, to December 8, 2021. Researchers trained in qualitative interviewing techniques from Adelphi Values conducted the interviews. The interviewers followed a semi-structured interview guide, and the interviews were audio-recorded after participants’ consent. Participants were asked to describe in their own words the experiences and impacts of bowel urgency and bowel urgency-related accidents. For example, participants were asked “Could you please tell me more about your experience with [participant’s term for bowel urgency] due to UC/CD?”; “How would you describe [participant’s term for bowel urgency]?”; “How often do you experience [participant’s term for bowel urgency]?”; and “How would you describe [participant’s term for bowel urgency] during a flare?” In addition, participants were asked about the aspects of bowel urgency experience that are most bothersome and how they define meaningful improvement in bowel urgency.

After the participants responded to the open-ended questions, targeted probes were used to elicit detailed descriptions (eg, participants’ description of a meaningful improvement in bowel urgency experience) and to obtain information on specific concepts of interest (eg, what the participants’ experience of abdominal pain with bowel urgency is like). Interviews typically lasted for approximately 90 minutes. After completion of interviews, the recordings were transcribed and anonymized. To organize and catalog the participants’ responses, the transcripts were coded and analyzed using qualitative data management software ATLAS.ti version 7 (ATLAS.ti Scientific Software Development).^38,39^

### Data Analysis

To assess the adequacy of the number of interviews conducted, concept saturation analyses were performed. Concept saturation reflects the number of interviews at which no new information is gathered by conducting additional interviews.^30,40,41^ Qualitative data generated from the CEIs were organized and used to evaluate the saturation of symptom concepts and impact domains experienced with bowel urgency and bowel urgency-related accidents in UC or CD. Based on the order of interviews, transcripts were divided into 4 groups (ie, 3-4 transcripts per group) for each disease group to develop saturation grids. Coded data were used to determine when a concept was mentioned for the first time in the interview, and the transcript group in which it was first mentioned was noted. Absence of the emergence of new concepts in the final transcript group indicated saturation of concept. Concept frequency describing the number of participants who reported experiencing a specific concept (eg, aspects of bowel urgency, co-occurring IBD symptoms, and impacts on HRQoL experienced in relation to bowel urgency or bowel urgency-related accidents) at least once during the occurrence of UC or CD was recorded and documented. In addition, concept clarification analyses, which included participant descriptions of concepts, were conducted to identify the salient features of the experience of bowel urgency, bowel urgency-related accidents, and the associated impacts. The symptom and impact concepts identified during the CEIs were presented as patient-centric conceptual models depicting the experience of bowel urgency and bowel urgency-related accidents in participants with UC or CD.

### Statistical Analysis

Continuous variables were presented as mean ± SD. Categorical variables were summarized as frequencies and percentages. The study was descriptive in nature, and no statistical comparisons were performed.

## Results

### CEIs: UC Group

#### Demographics

Fifteen participants with moderate-to-severe UC were recruited from 5 clinical sites in the United States. Females comprised 53.3% (n = 8 of 15) of the study population. The mean age of the participants was 51.6 ± 19.8 years, and a majority were White (80%, n = 12 of 15) (Table 1). Overall, 73.3% of participants were reported by clinicians to have moderate UC, and 26.7% had severe UC. Overall, 60.0% of participants reported experiencing bowel urgency in the past week, and 33.3% experienced bowel urgency-related accidents in the past 4 weeks (Table 1).

**Table 1.** Patient demographics and baseline clinical characteristics

Characteristic	UC (n = 15)	CD (n = 15)
Patient-reported information^a^		
Age, y		
Range	18.8–85.1	39.3–75.3
Mean ± SD	51.6 ± 19.8	50.0 ± 16.8
Biological sex		
Female	8 (53.3)	8 (53.3)
Male	7 (46.7)	7 (46.7)
Ethnicity		
Mexican/Mexican American, Chicano	2 (13.3)	3 (20.0)
Not Spanish/Hispanic/Latino	13 (86.7)	12 (80.0)
Race		
American Indian or Alaska Native	1 (6.7)	0 (0.0)
Black or African American	2 (13.3)	6 (40.0)
Hispanic	0 (0.0)	1 (6.7)
White	12 (80.0)	8 (53.3)
Current living situation		
Living with family or friends (roommate, parent, child, partner/spouse)	9 (60.0)	9 (60.0)
Living alone	6 (40.0)	6 (40.0)
Current work status		
Working full-time	4 (26.7)	10 (66.7)
Retired	4 (26.7)	3 (20.0)
Student	2 (13.3)	1 (6.7)
Unemployed	2 (13.3)	0 (0.0)
Working part-time	1 (6.7)	1 (6.7)
Homemaker	1 (6.7)	0 (0.0)
On disability	1 (6.7)	0 (0.0)
Highest level of education		
High school diploma (or GED) or less	4 (26.7)	7 (46.7)
Some college or certificate program	7 (46.7)	1 (6.7)
College or university degree (2 or 4 y)	1 (6.7)	6 (40.0)
Graduate degree	3 (20.0)	1 (6.7)
Frequency of bowel urgency in the last week		
Have not experienced bowel urgency in the last week or less than once a week	6 (40.0)	8 (53.3)
Once or twice a week	4 (26.7)	3 (20.0)
Several times a week	0 (0.0)	3 (20.0)
Once or twice a day	2 (13.3)	1 (6.7)
Several times a day	3 (20.0)	0 (0.0)
Frequency of bowel urgency-related accidents in the past 4 weeks		
Have not experienced an accident in the past 4 weeks	10 (66.7)	12 (80.0)
1–4 accidents in the past 4 weeks	3 (20.0)	3 (20.0)
5–8 accidents in the past 4 weeks	1 (6.7)	0 (0.0)
More than 8 accidents in the past 4 weeks	1 (6.7)	0 (0.0)
Other health conditions		
None	6 (40.0)	4 (26.7)
High blood pressure	2 (13.3)	5 (33.3)
High cholesterol	1 (6.7)	5 (33.3)
Migraine headaches	2 (13.3)	4 (26.7)
Other^a^	3 (20.0)	2 (13.3)
Stomach/intestinal disorder	4 (26.7)	0 (0.0)
Depression/anxiety	2 (13.3)	1 (6.7)
Diabetes	3 (20.0)	2 (13.3)
Diabetes (type 1)	0 (0.0)	0 (0.0)
Diabetes (type 2)	3 (20.0)	1 (6.7)
Rheumatoid arthritis	1 (6.7)	2 (13.3)
Asthma	1 (6.7)	0 (0.0)
Cancer	1 (6.7)	0 (0.0)
Heart disease	1 (6.7)	0 (0.0)
Fibromyalgia	1 (6.7)	0 (0.0)
Kidney disorder	0 (0.0)	1 (6.7)
Thyroid disease	1 (6.7)	0 (0.0)
Clinician-reported information		
Phenotype (for CD participants only)		
Small bowel involvement only, including isolated ileitis	—	5 (33.3)
Colonic involvement, with or without small bowel involvement (proximal ± transverse colon only)	—	2 (13.3)
Colonic involvement, with or without small bowel involvement (rectal only)	—	2 (13.3)
Colonic involvement, with or without small bowel involvement (rectal + distal colon only)	—	4 (26.7)
Colonic involvement, with or without small bowel involvement (involvement of all colonic segments)	—	2 (13.3)
Biologic/conventional UC or CD therapy experience		
Yes—participant is on biologic and/or conventional therapy	12 (80.0)	13 (86.7)
No—participant is not on biologic and/or conventional therapy	3 (20.0)	2 (13.3)
UC/CD severity (as rated on PGA)		
Moderate disease	11 (73.3)	8 (53.3)
Severe disease	4 (26.7)	7 (46.7)

Values are n (%), unless otherwise indicated. Percentages were calculated based on the total number of participants in each group.

Abbreviations: CD, Crohn’s disease; PGA, Physician Global Assessment; UC, ulcerative colitis.

^a^Participant report of “other” conditions included chronic obstructive pulmonary disease, psoriasis, hiatal hernia, Sjogren’s syndrome, dry eye disease, and hearing loss.

#### Saturation of concepts

##### Saturation of sign and symptom concepts

Saturation analysis of the sign, symptom, and impact domain concepts elicited from participants with UC is described in Supplementary Table 1. Thirteen bowel urgency-related sign and symptom concepts were spontaneously reported by the participants, all of which were elicited prior to the final group of interviews (ie, transcript group 4; n = 3). No new concepts emerged in the final group of interviews, indicating that saturation of sign and symptom concepts co-occurring with the experience of bowel urgency in the UC group was achieved. Six sign and symptom concepts associated with bowel urgency-related accidents were spontaneously reported. Two new concepts, gas and fatigue, emerged in the final group of interviews from 1 participant. This participant had first reported experiencing gas and fatigue as part of bowel urgency experience, and subsequently confirmed that these were also part of the bowel urgency-related accident experience.

##### Saturation of impact domains

Sixteen impact domains associated with the experience of bowel urgency were spontaneously reported by the participants. All impact domains were reported prior to the final group of interviews, indicating that saturation of impact domains of bowel urgency in the UC group was achieved. Thirteen impact domains associated with the experience of bowel urgency-related accidents were spontaneously reported by the participants. Of these, the domain “self-image” emerged in the final group of interviews.

For participants with UC, saturation was achieved for most sign and symptom concepts and impact domains that were elicited prior to the final group of interviews. The signs, symptoms, and impact domains that emerged in the final group of interviews differed substantially from those reported in the prior interview groups; therefore, it cannot be concluded that saturation was achieved for all bowel urgency-related accident concepts. However, as sign and symptom concepts and impacts occurring with the experience of bowel urgency-related accidents were not the primary focus of this study, no additional interviews were deemed necessary.

#### Evaluation of concept clarification and frequency

Patient-reported descriptions of severity, frequency, duration, and occurrence of bowel urgency and bowel urgency-related accidents are summarized as concept description tables (Supplementary Tables 2 and 3). Frequencies and descriptions of unique concepts of signs, symptoms, and impacts of bowel urgency and bowel urgency-related accidents are presented in Supplementary Tables 4 and 5. The frequency with which the concepts are reported as the most bothersome aspects and the frequency with which a decrease in the symptom was reported as a meaningful improvement are summarized in Tables 2 and 3.

**Table 2.** Patient-reported aspects of bowel urgency that are most bothersome and meaningful to improve

Concepts reported by participants	UC (n = 15)		CD (n = 15)	
	Most bothersome aspect of bowel urgency^a^	Improvement in this concept indicates meaningful improvement in bowel urgency^a^	Most bothersome aspect of bowel urgency^a^	Improvement in this concept indicates meaningful improvement in bowel urgency^a,b^
IBD signs and symptoms experienced with bowel urgency				
Abdominal pain^c,d^	6	4	3	3
Fatigue	–	1	–	0
Abdominal cramping^c,d^	6	1	2	0
Diarrhea	1	0	0	1
Nausea	0	–	1	–
Impacts experienced with bowel urgency				
Adaptive behaviors	n = 15		n = 14	
Bathroom mapping	3	–	0	–
Use of protective undergarments	2	–	0	–
Emotional functioning	n = 13		n = 12	
Stress	0	–	1	–
Frustration	–	0	–	1
Worry	1	2	0	1
Social functioning	n = 10		n = 13	
Inability to participate in social activities	4	2	4	4
Recreational/leisure activities	n = 6		n = 12	
Difficulty engaging in hobbies or leisure activities	0	–	2	–
Sleep	n = 8		n = 10	
Interrupted sleep	0	0	2	2
Work	n = 7		n = 10	
Interrupted work activities	0	0	3	1
Family activities	n = 4		n = 7	
Inability to participate in family activities	0	0	3	1
Sexual health	n = 5		n = 5	
Impacted sexual activity	1	1	1	1
Activities of daily living	n = 3		–	
Interrupted eating	–	1	–	0

Abbreviations: CD, Crohn’s disease; IBD, inflammatory bowel disease; UC, ulcerative colitis.

^a^Counts are not mutually exclusive.

^b^One participant (CD) reported that all impacts of bowel urgency would be important to improve.

^c^UC: 4 participants reported abdominal pain and abdominal cramping to be the same, 10 reported abdominal pain and abdominal cramping to be different.

^d^CD: 4 participants reported abdominal cramping and abdominal pain to be the same, 6 reported abdominal pain and abdominal cramping to be different.

**Table 3.** Patient-reported aspects of bowel urgency-related accidents that are most bothersome and meaningful to improve

Concept reported by participants	UC (n = 13)		CD (n = 15)	
	Most bothersome aspect of bowel urgency-related accidents^a^	Improvement in this concept indicates meaningful improvement in bowel urgency-related accidents^*,‡^	Most bothersome aspect of bowel urgency-related accidents^a^	Improvement in this concept indicates meaningful improvement in bowel urgency-related accidents^a^
IBD signs and symptoms experienced bowel urgency-related accidents				
Abdominal pain	–	1	–	1
Impacts experienced with bowel urgency-related accidents				
Adaptive behaviors	n = 13		n = 15	
Dietary changes	–	1	–	0
Use of protective undergarments	–	1	–	0
Ensuring adequate supplies/extra change of clothes	1	1	0	0
Emotional functioning	n = 10		n = 13	
Embarrassment	3	0	2	1
Fear	1	1	0	0
Stress	0	0	1	1
Worry	1	–	0	–
Frustration	–	0	–	1
Social functioning	n = 8		n = 10	
Inability to participate in social activities	3	3	1	0
Social isolation	–	0	–	1
Changing plans	0	0	1	1
Travel	n = 7		n = 9	
Avoid or delay travel	1	–	0	–
Inability to travel	–	0	–	1
Independence	n = 1		n = 7	
Lack of control over daily life	–	0	–	1
Work	n = 2		n = 6	
Interrupted work activities	0	–	1	–
Sleep	n = 2		n = 5	
Interrupted sleep	1		0	
Family activities	n = 0		n = 3	
Inability to participate in family activities	0	–	2	–

Abbreviations: CD, Crohn’s disease; IBD, inflammatory bowel disease; UC, ulcerative colitis.

^a^Counts are not mutually exclusive.

##### Experience of bowel urgency

All participants with UC experienced bowel urgency and described it as a strong or sudden need to use the bathroom for a bowel movement, often to an extent that they are unable to wait to have a bowel movement. Participants defined bowel urgency severity as the frequency of bowel movements or needing to return to bathroom due to unfinished bowel movements, how urgently or quickly one needs to go to the bathroom, the amount of pain and/or cramping, the lack of control over urgency, the amount of discharge or diarrhea during a bowel movement, how tired one gets when urgency occurs, the consistency of stool, and whether accidents occur (Supplementary Table 2).

Abdominal pain or abdominal cramps (n = 6) and frequency of bowel urgency (n = 3) were reported as the most bothersome aspects of bowel urgency experience (Supplementary Table 2). Participants described decreased frequency of bowel urgency (n = 5), abdominal pain (n = 4), decrease in the severity of bowel urgency (n = 4), and improved control (n = 3) as the aspects of bowel urgency that were meaningful to improve.

##### Bowel urgency-related signs, symptoms, and impacts

Participants with UC spontaneously reported 13 sign and symptom concepts occurring with the experience of bowel urgency, with abdominal pain (n = 13 [86.7%]), fatigue (n = 12 [80.0%]), and abdominal cramping (n = 10 [66.7%]) being the most frequently reported (Supplementary Table 4). One additional concept, loss of appetite, was reported via probed questioning by 1 (6.7%) participant. Overall, 32 bowel urgency-related impact concepts across 16 domains were reported. Adaptive behaviors (n = 15 [100%]), emotional functioning (n = 13 [86.7%]), and social functioning (n = 10 [66.7%]) were the most frequently reported impact domains. The most frequently reported impacts were dietary changes (n = 11 [73.3%]), inability to participate in social activities (n = 10 [66.7%]), and difficulty running errands (n = 9 [60.0%]). The impacts reported as most bothersome to participants included inability to participate in social activities (n = 4) and bathroom mapping (n = 3) (Table 2). Inability to participate in social activities (n = 2) and worry (n = 2) were the impacts that were most meaningful to improve.

##### Experience of bowel urgency-related accidents

Thirteen (86.7%) participants with UC reported experiencing bowel urgency-related accidents. The incidence of bowel urgency-related accidents was described as the inability of participants to make it to the bathroom when experiencing bowel urgency or experiencing a situation wherein they had little or no control of their bowels (Supplementary Table 3). These accidents occurred spontaneously with little to no warning and were likely to occur more frequently during a disease flare-up. Eight participants experienced flare-related accidents. Accidents that occurred due to flare-ups were reported to be more severe or intense, with runnier stools, and participants having less time to visit the restroom. Participants were not asked to report on what aspect of bowel urgency-related accidents was most bothersome. Improvements that were most meaningful included complete absence of bowel urgency-related accidents (n = 4).

##### Signs, symptoms, and impacts associated with bowel urgency-related accidents

Participants reported 7 sign and symptom concepts that occur with bowel urgency-related accidents, with abdominal pain (n = 10 [76.9%]), gas (n = 10 [76.9%]), and abdominal cramping (n = 9 [69.2%]) being the most frequently reported (Supplementary Table 5). Overall, 30 impacts of bowel urgency-related accidents were reported by participants across 13 domains. Adaptive behaviors (n = 13 [100%]), emotional functioning (n = 10 [76.9%]), and social functioning (n = 8 [61.5%]) were the most frequently reported domains. Bathroom mapping (n = 8 [61.5%]), inability to participate in social activities (n = 8 [61.5%]), and embarrassment (n = 7 [53.8%]) were the most frequently reported impacts. Impacts that were most bothersome for participants were inability to participate in social activities (n = 3) and embarrassment (n = 3); inability to participate in social activities (n = 3) was the most important to improve (Table 3).

### CEIs: CD Group

#### Demographics

Fifteen participants with CD were recruited from 4 clinical sites in the United States. Of these, 5 participants had a phenotype of small bowel involvement only (including isolated ileitis); 4 had rectal with distal colon involvement; and 2 each had proximal with transverse colon involvement, only rectal involvement, and involvement of all colonic segments. Females comprised 53.3% (n = 8 of 15) of the population, and the mean age of the participants was 50.0 ± 16.8 years (Table 1). A total of 53.3% of participants were reported by their clinicians to have moderate CD, and 46.7% had severe CD. In the past week, 53.3% of participants reported experiencing bowel urgency, and 26.7% experienced bowel urgency-related accidents in the past 4 weeks.

#### Saturation of concepts

Saturation analyses of the sign, symptom, and impact domain concepts elicited from participants with CD are described in Supplementary Table 6.

##### Saturation of sign and symptom concepts

Fifteen and 11 sign and symptom concepts associated with bowel urgency and bowel urgency-related accidents, respectively, were reported spontaneously by the CD group. All sign and symptom concepts were reported prior to the final group of interviews, indicating that saturation of sign and symptoms concepts had been achieved.

##### Saturation of impact domains

Sixteen impact domains associated with the experience of bowel urgency were spontaneously reported by the CD group. One participant reported an HRQoL domain, “spouse/partner relationships,” in the final group of interviews. In relation to the experience of bowel urgency-related accidents, 16 impact domains were spontaneously reported. Two (13.3%) participants reported an HRQoL domain, “spouse/partner relationships,” in the final group of interviews.

For participants with CD, saturation was achieved for most sign and symptom concepts and impact domains that were elicited prior to the final group of interviews. The impact domains that emerged in the final group of interviews differed substantially from those reported in the prior interview groups; therefore, it cannot be concluded that saturation was achieved for all bowel urgency-related accident concepts. However, as sign and symptom concepts and impacts occurring with the experience of bowel urgency-related accidents were not the primary focus of this study, no additional interviews were deemed necessary.

#### Evaluation of concepts clarification and frequency

Patient-reported descriptions of severity, frequency, duration, and occurrence of bowel urgency and bowel urgency-related accidents are summarized as concept description tables (Supplementary Tables 2 and 3). Frequencies and descriptions of unique concepts of signs, symptoms, and impacts of bowel urgency and bowel urgency-related accidents reported by the participants are presented in Supplementary Tables 4 and 5. The frequency with which the concepts are reported as the most bothersome aspects and the frequency with which a decrease in the symptom was reported as a meaningful improvement are summarized in Tables 2 and 3.

##### Experience of bowel urgency

All participants with CD experienced bowel urgency and described it as a strong or sudden need to use the bathroom for a bowel movement, often to an extent that they are unable to wait to have a bowel movement. Participants described bowel urgency severity as the amount of time that they have or not being able to wait at all before needing to use the restroom, the frequency or duration of bowel urgency, increased and uncontrolled symptomology associated with bowel urgency (abdominal pain, overall symptoms, irritation, constipation), having a flare-up, dependent on the severity of the resulting bowel movement, and accidents (Supplementary Table 2). The urgent need to use or be near the bathroom (n = 2), uncertainty of the condition (n = 2), fear of experiencing an accident (n = 2), and all aspects of bowel urgency (n = 3) were found to be the most bothersome aspects of bowel urgency experience. Bowel urgency frequency (n = 5), complete resolution/reduced bathroom visits due to bowel urgency (n = 4), abdominal pain (n = 3), and having more time to get to the bathroom (n = 3) were reported as the aspects of bowel urgency that were meaningful to improve (Supplementary Table 2).

##### Bowel urgency-related sign, symptom, and impact concepts

Participants with CD reported 16 sign and symptom concepts that occur with bowel urgency, with abdominal pain and fatigue (n = 13 each [86.7%]) and abdominal cramping (n = 8 [53.3%]) being the most frequently reported (Supplementary Table 4). A total of 36 bowel urgency-related impact concepts were reported by participants across 16 domains. Most frequently reported impact domains were adaptive behaviors (n = 14 [93.3%]), household chores and social activities (n = 13 each [86.7%]), and emotional functioning and recreational/leisure activities (n = 12 each [80.0%]). Impact concepts reported most frequently were dietary changes (n = 13 [86.7%]), inability to participate in social activities (n = 13 [86.7%]), interrupted sleep (n = 10 [66.7%]), and bathroom mapping (n = 8 [53.3%]). The impacts reported as most bothersome to participants included inability to participate in social activities (n = 4), interrupted work activities (n = 3), and inability to participate in family activities (n = 3) (Table 2). Inability to participate in social activities (n = 4) and interrupted sleep (n = 2) were the impacts that were most meaningful to improve.

##### Experience of bowel urgency-related accidents

All participants with CD reported experiencing bowel urgency-related accidents. The incidence of bowel urgency-related accidents was described as the inability of participants to make it to the bathroom when experiencing bowel urgency or experiencing a situation wherein they had little or no control of their bowels (Supplementary Table 3). These accidents occurred spontaneously with little to no warning and may occur more frequently during a disease flare-up. In the CD group, 8 (53.3%) participants experienced flare-related accidents, with 3 (20%) participants experiencing accidents only during flare-ups. Accidents that occurred due to flare-ups were reported to be more severe or intense, with runnier stools, and participants having less time to visit the restroom. Participants described feeling a stronger “rush”; feeling “faster” or more “sudden” urgency; experiencing pain and cramping; and having a stronger stomach bubbling and diarrhea sensation, with accidents occurring in the middle of night. Participants were not asked to report on what aspect of bowel urgency-related accidents was most bothersome. Improvements that were most meaningful included complete absence of bowel urgency-related accidents (n = 3) and decreased bowel urgency (n = 3) (Supplementary Table 3).

##### Signs, symptoms, and impacts associated with bowel urgency-related accidents

Twelve sign and symptom concepts occurring with bowel urgency-related accidents were reported, with abdominal pain (n = 10 [66.7%]), fatigue (n = 10 [66.7%]), abdominal cramping (n = 9 [60.0%]), loss of appetite (n = 8 [53.3%]), and mucus in stool (n = 8 [53.3%]) being the most frequent (Supplementary Table 5). Overall, 44 impact concepts associated with bowel urgency-related accidents across 16 domains were reported. The most frequently reported domains were adaptive behaviors (n = 15 [100%]), emotional functioning (n = 13 [86.7%]), social functioning (n = 10 [66.7%]), household chores (n = 10 [66.7%]), recreational/leisure activities (n = 10 [66.7%]), and travel (n = 8 [53.3%]). Dietary changes (n = 12 [80.0%]), embarrassment (n = 9 [60.0%]), and social isolation (n = 8 [53.3%]) were the most frequently reported impacts. All impacts (n = 2), inability to participate in family activities (n = 2), and embarrassment (n = 2) were most frequently reported as most bothersome (Table 3). Changing plans, embarrassment, frustration, inability to travel, lack of control over daily life, social isolation, and stress (n = 1 each) were most important to improve.

#### Patient-centric conceptual models

**Figure 1.**
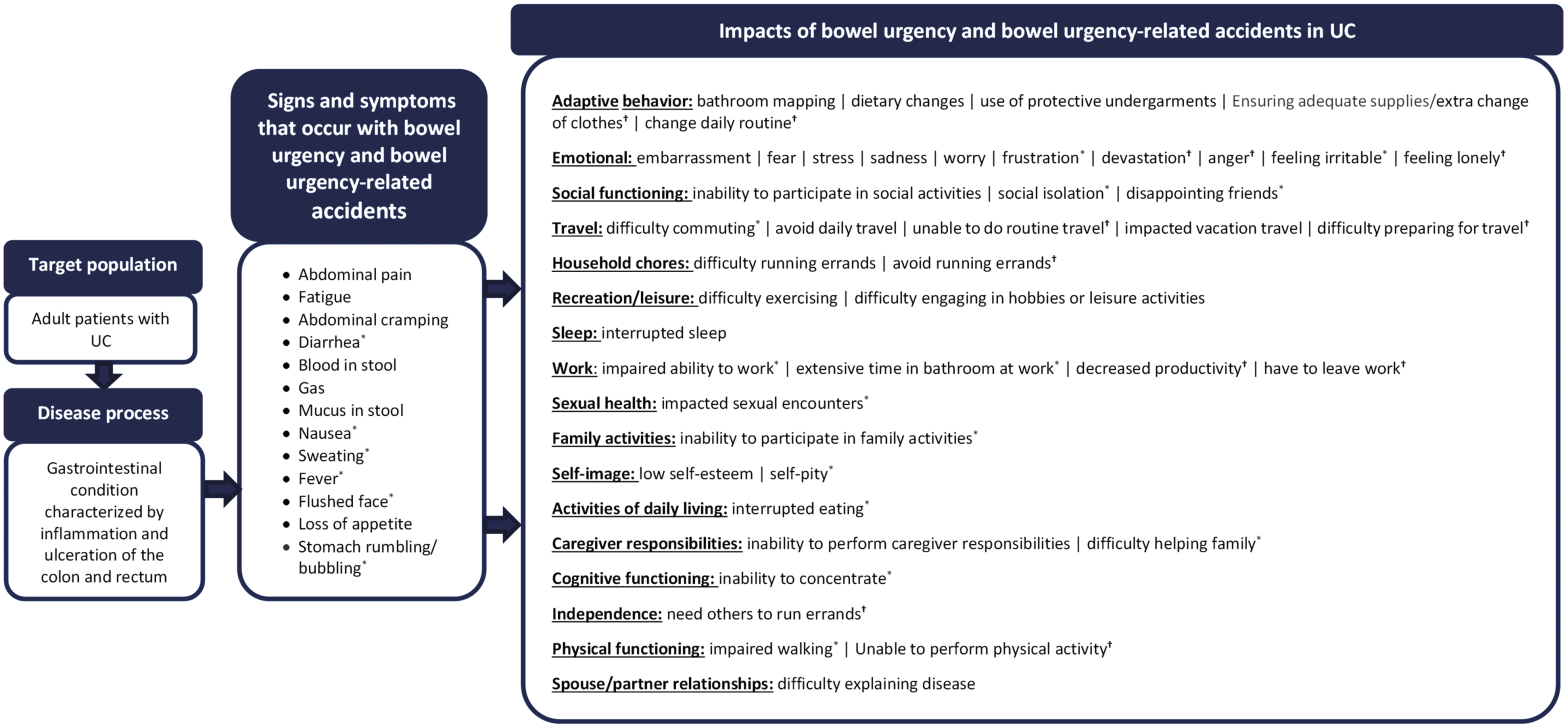
Patient-centric conceptual model of bowel urgency and bowel urgency-related accidents associated with ulcerative colitis (UC). Sign and symptom concepts are presented in order of frequency of report (ie, most to least frequent). Impacts are presented in order of most frequently reported domain followed by impact concepts within each domain, which are presented in order of frequency of report. ^*^Concepts were only reported by participants for bowel urgency. ^†^Concepts were only reported by participants for bowel urgency-related accidents.

**Figure 2.**
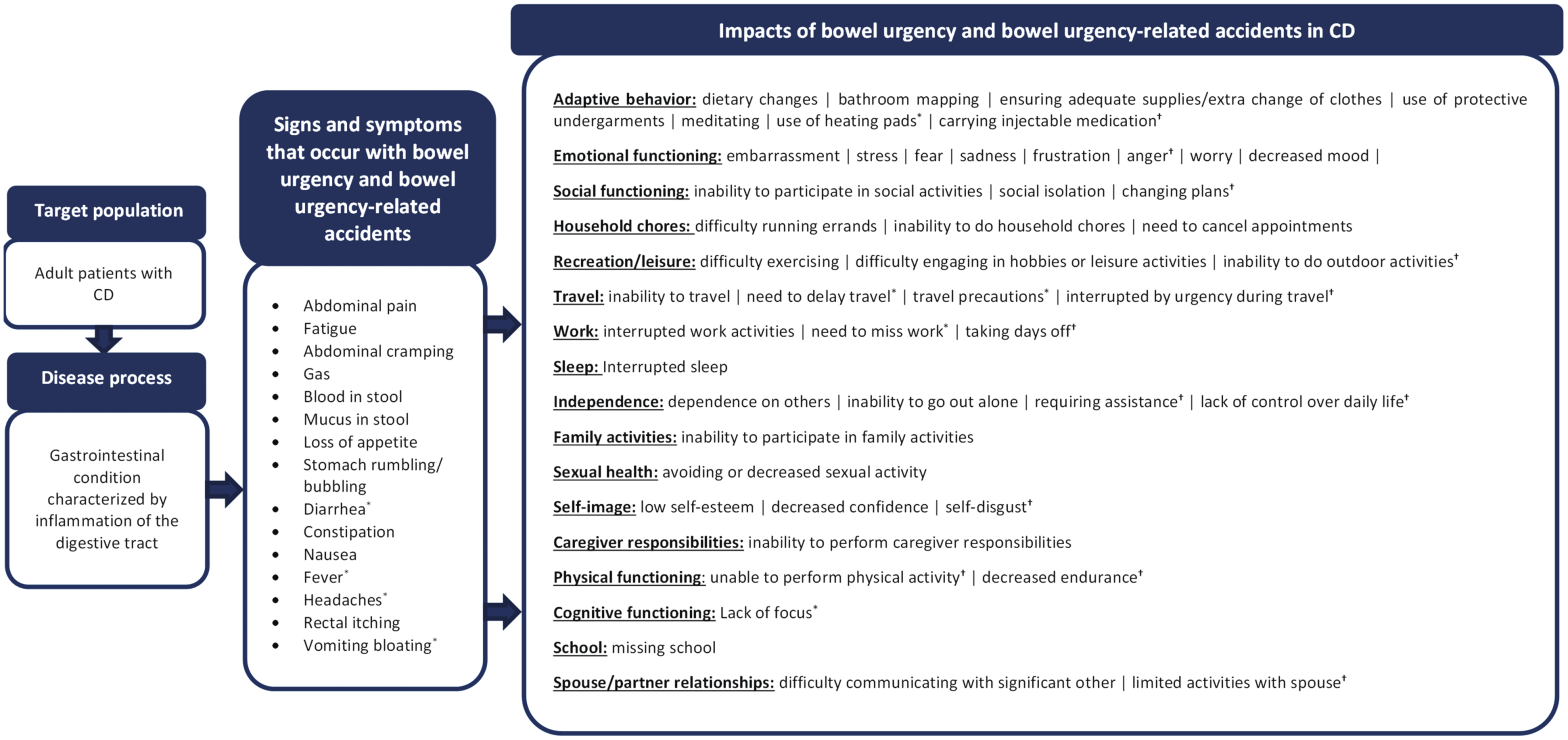
Patient-centric conceptual model of bowel urgency and bowel urgency-related accidents associated with Crohn’s disease (CD). Sign and symptom concepts are presented in order of frequency of report (ie, most to least frequent). Impacts are presented in order of most frequently reported domain followed by impact concepts within each domain, which are presented in order of frequency of report. ^*^Concepts were only reported by participants for bowel urgency. ^†^Concepts were only reported by participants for bowel urgency-related accidents.

To understand the sign, symptom, and impact concepts associated with bowel urgency and bowel urgency-related accidents, conceptual models were developed for UC and CD. All the sign and symptom concepts and impact domains associated with bowel urgency and bowel urgency-related accidents identified for UC and CD were organized into patient-centric conceptual models (Figures 1 and 2). The models present every reported impact and its corresponding domain associated with bowel urgency and bowel urgency-related accidents, showing a total of 17 impact domains and 45 impact concepts for UC and 17 impact domains and 48 impact concepts for CD.

## Discussion

Previous survey-based studies have reported that among the diverse symptoms experienced by patients with UC or CD, bowel urgency and bowel urgency-related accidents are bothersome and impact patients’ overall well-being.^6,7,42^ The present study built on these findings by qualitatively exploring the experience of bowel urgency and bowel urgency-related accidents and which aspects of these experiences were most bothersome and meaningful to improve in patients.

All participants with UC or CD reported experiencing bowel urgency in the past 3 months, and all except 2 participants with UC, reported experiencing bowel urgency-related accidents. In agreement with previous reports, our findings suggested that the experience of bowel urgency and bowel urgency-related accidents was bothersome and affected the HRQoL of patients.^6,7,26^ UC and CD have similar disease burden,^43^ which was evident in our study as the symptoms and impacts on HRQoL experienced due to bowel urgency and bowel urgency-related accidents were similar among participants with UC and CD. In concordance with the findings of Dubinsky et al^42^ suggesting that bowel urgency is not experienced in isolation, participants with UC or CD reported several co-occurring symptoms. The most frequently reported symptoms co-occurring with bowel urgency were abdominal pain, abdominal cramping, and fatigue, while those related to bowel urgency-related accidents were abdominal pain and abdominal cramping.

In both groups, decreased frequency of bowel urgency was reported as the most meaningful aspect to improve, and a decrease in abdominal pain was also described as a meaningful improvement. Furthermore, not wanting to experience bowel urgency-related accidents at all was most frequently reported as a meaningful improvement in the bowel urgency-related accident experience.

The changes to daily activities and emotional and mental burden of managing bowel urgency to avoid bowel urgency-related accidents described by participants in this study indicate the significant HRQoL impact of bowel urgency on patients’ lives. Across both groups, the most frequently reported impact concepts of bowel urgency were dietary changes, difficulty running errands, and inability to participate in social activities. Among participants with UC, bathroom mapping and inability to participate in social activities were frequently reported impacts of bowel urgency-related accidents, while dietary changes and social isolation were reported by participants with CD. Previous studies have highlighted the attitudes and perceptions related to changes in dietary behavior, dietary beliefs, and eating disorders and the associated psychosocial distress in patients with IBD.^44-46^ In agreement with these reports, in this study, “dietary changes” was the most frequently reported impact of bowel urgency among participants with UC (73.3%) or CD (86.7%).

The psychological and emotional impacts associated with bowel urgency and bowel urgency-related accidents have been reported to be devastating in patients with UC or CD.^7,47^ Among these impacts, feeling of embarrassment is commonly experienced by patients^48^ and is a major cause of underreporting of symptoms.^49^ Similar to these reports, embarrassment was the most frequently reported accident impact concept across both groups in this study.

Patients have reported that bathroom mapping, the need to use the bathroom shortly after eating, and finding a bathroom during bowel urgency considerably impact the HRQoL of patients and impose a significant psychosocial burden.^12,16,50^ In agreement with these findings, bathroom mapping was reported as an impact of bowel urgency and bowel urgency-related accidents both in participants with UC and CD. Participants described bathroom mapping as being aware of bathroom locations and being near bathrooms to avoid any bowel urgency-related accidents.

The occurrence and severity of bowel urgency and bowel urgency-related accidents in patients with UC are associated with disease flare-ups.^31,51,52^ Interview findings suggested that a majority of participants with UC and CD experienced bowel urgency-related accidents as part of disease flare-ups, and 20% of participants with CD reported experiencing bowel urgency only during a flare-up.

When defining bowel urgency severity, patients think about the frequency of bowel urgency and other factors such as duration of bowel urgency, how much time they have to get to the bathroom before a bowel movement, the severity of co-occurring IBD symptoms, and whether accidents occur. These findings highlight the multidimensional nature of bowel urgency, indicating that multiple aspects of bowel urgency must be considered when developing bowel urgency–specific PRO measures. To our knowledge, this is the first study to capture the patients’ perspective of the multidimensional aspects of bowel urgency. Patient-centric conceptual models, developed based on information from the CEIs, provide a comprehensive overview of the signs, symptoms, and impacts of bowel urgency and their interrelationships, facilitating the identification of focus areas for developing treatment strategies and organizing specific concepts to develop PRO questionnaires. Based on the identified concepts, future quantitative studies evaluating the content validity of PRO measures (psychometric analyses and score interpretability) for clinical use as a metric of disease activity are needed.

### Limitations

A few limitations must be considered when interpreting the results of this study. First, participants were initially asked to describe their experiences of bowel urgency followed by those of bowel urgency-related accidents, which might have caused an overlap in the reported experiences. This resulted in the emergence of fewer new concepts during exploration of bowel urgency-related accidents. Nevertheless, participants confirmed that experiences of bowel urgency and bowel urgency-related accidents were closely related and often similar. Second, when evaluating saturation, if concept saturation is not achieved, additional interviews may be considered to ensure that the sample size was adequate to elicit the most important and relevant concepts for each condition. In this study, though saturation of concept was not achieved for bowel urgency-related accidents, the key concepts co-occurring with bowel urgency and bowel urgency-related accidents had emerged in the sample that was interviewed, and additional interviews were not needed. Third, clinical trials and clinical outcome assessment studies are actively working toward more inclusive research and better representation of participants. A few characteristics of the interview study sample were skewed (eg, 80% of UC participants were White) and did not overrepresent the historically underrepresented racial and ethnic groups. Therefore, the findings may not be generalized to represent the perspectives of the broad IBD population, and further research among more representative and diverse populations is needed. Fourth, the participants were not asked if they had a diagnosis of concomitant irritable bowel syndrome, the presence of which may influence the participants’ responses. Last, the study did not include a quantitative analysis to compare UC and CD subgroups. Thus, further research is needed to understand the similarities and differences in the experiences of bowel urgency and bowel urgency-related accidents between UC and CD patients.

## Conclusions

The symptom concepts and impact domains associated with the experiences of bowel urgency and bowel urgency-related accidents were similar across UC and CD and were bothersome and impactful on patients’ daily lives. The results highlight the need for development of novel treatment strategies to reduce the frequency of bowel urgency and to prevent bowel urgency-related accidents. Therefore, assessment of bowel urgency and bowel urgency-related accidents must be considered in clinical trials and by healthcare providers in routine clinical examinations. Our findings may facilitate the development of appropriate PRO measures to assess and discuss bowel urgency in clinical practice and clinical trials, manage symptoms, and disseminate the impacts of treatments on disease activity and severity.

## Supplementary data

Supplementary data is available at *Inflammatory Bowel Diseases* online.

izae044_suppl_Supplementary_Material

## Data Availability

The datasets analyzed during the current study are not publicly available due to proprietary reasons and are intellectual property of Eli Lilly and Company.
