# Supplementary material for: Patient Perspectives of Bowel Urgency and Bowel Urgency-Related Accidents in Ulcerative Colitis and Crohn’s Disease
Source: Inflamm Bowel Dis. 2024 Mar 21;30(10):1831–42. doi: 10.1093/ibd/izae044 (PMC11447055; doi:10.1093/ibd/izae044)
Supplement: izae044_suppl_Supplementary_Material [file izae044_suppl_supplementary_material.docx]

**Supplementary Information**

**Patient Perspectives of Bowel Urgency and Bowel Urgency-Related Accidents in Ulcerative Colitis and Crohn’s Disease**

Vipul Jairath^1^, MBChB, DPhil, Theresa Hunter Gibble^2^, PhD, Alison Potts Bleakman^2^, PhD, Kaitlin Chatterton^3^, MPH, Paolo Medrano^3^, MA, Megan McLafferty^3†^, MA, Brittany Klooster^3^, MPH, Sonal Saxena^2^, PhD, Richard Moses^2^, DO JD

^1^Department of Medicine, Division of Gastroenterology, Western University, London, Ontario, OX3 9DU, Canada.

^2^Eli Lilly and Company, Indianapolis, USA

^3^Adelphi Values, Patient Centered Outcomes, Boston, Massachusetts, USA

^†^Author affiliation has changed since the time this research was conducted. Assigned affiliation is the institution of employment at the time this research was conducted.

### Corresponding author:

Theresa Hunter Gibble

Eli Lilly and Company

893 S. Delaware Street

Indianapolis, IN 46225, USA

Phone: +1 3176510341

E-mail: [hunter_theresa_marie@lilly.com](mailto:hunter_theresa_marie@lilly.com)

**Supplementary Figure 1.** **Overview of Research Methodology to Explore Ulcerative Colitis and Crohn’s Disease Patient Experience of Bowel Urgency and Bowel Urgency-Related Accidents**

CD, Crohn’s disease; UC, ulcerative colitis

**Supplementary Table 1. Saturation Grid for the Ulcerative Colitis Group**

| Root concept or domain (n) | UC participants (N=15) | | | |
| --- | --- | --- | --- | --- |
|  | Transcript group 1^†^ (n=4) | Transcript group 2^‡^ (n=4) | Transcript group 3^§^ (n=4) | Transcript group 4^**^ (n=3) |
| Bowel urgency | | | | |
| Sign and symptom concepts occurring with the experience of bowel urgency | | | | |
| Abdominal pain (n=12) | X |  |  |  |
| Abdominal cramping (n=10) | X |  |  |  |
| Diarrhea (n=4) | X |  |  |  |
| Fatigue (n=4) | X |  |  |  |
| Gas (n=4) | X |  |  |  |
| Blood in stool (n=2) | X |  |  |  |
| Nausea (n=2) | X |  |  |  |
| Sweating (n=2) |  | X |  |  |
| Constipation (n=1) | X |  |  |  |
| Fever (n=1) |  | X |  |  |
| Flushed face (n=1) | X |  |  |  |
| Mucus in stool (n=1) | X |  |  |  |
| Stomach rumbling/bubbling (n=1) |  | X |  |  |
| Domains of impacts associated with experience of bowel urgency | | | | |
| Adaptive behaviors (n=15) | X |  |  |  |
| Emotional functioning (n=13) | X |  |  |  |
| Social functioning (n=10) | X |  |  |  |
| Household chores (n=9) | X |  |  |  |
| Travel (n=9) | X |  |  |  |
| Sleep (n=8) | X |  |  |  |
| Work (n=7) |  | X |  |  |
| Recreational/leisure activities (n=6) | X |  |  |  |
| Sexual health (n=5) | X |  |  |  |
| Family activities (n=4) |  | X |  |  |
| Activities of daily living (n=3) | X |  |  |  |
| Self-image (n=3) | X |  |  |  |
| Caregiver responsibilities (n=2) |  | X |  |  |
| Cognitive functioning (n=2) | X |  |  |  |
| Physical functioning (n=1) | X |  |  |  |
| Spouse/partner relationships (n=1) |  |  | X |  |
| Bowel urgency-related accidents | | | | |
| Sign and symptom concepts occurring with the experience of bowel urgency-related accidents | | | | |
| Abdominal pain (n=9) | X |  |  |  |
| Abdominal cramping (n=8) | X |  |  |  |
| Blood in stool (n=2) | X |  |  |  |
| Fatigue (n=1) |  |  |  | X |
| Gas (n=1) |  |  |  | X |
| Loss of appetite (n=1) | X |  |  |  |
| Domains of impacts associated with the experience of bowel urgency-related accidents | | | | |
| Adaptive behaviors (n=13) | X |  |  |  |
| Emotional functioning (n=10) | X |  |  |  |
| Social activities (n=8) | X |  |  |  |
| Travel (n=7) | X |  |  |  |
| Household chores (n=4) | X |  |  |  |
| Recreational/leisure activities (n=4) | X |  |  |  |
| Physical functioning (n=2) | X |  |  |  |
| Sleep (n=2) | X |  |  |  |
| Work (n=2) |  | X |  |  |
| Caregiver responsibilities (n=1) |  | X |  |  |
| Independence (n=1) |  |  | X |  |
| Self-image (n=1) |  |  |  | X |
| Spouse/partner relationships (n=1) |  |  | X |  |

N= Total number of participants in each group

n=Number of spontaneous concept or domain reports

UC: ulcerative colitis

^†^Participants included in transcript group 1: 40-03, 70-01, 70-02, 70-03

^‡^Participants included in transcript group 2: 30-01, 30-02, 40-05, 40-06

^§^Participants included in transcript group 3: 20-01, 20-05, 30-03, 90-06

^**^Participants included in transcript group 4: 70-04, 90-02, 90-05

**Supplementary Table 2. Patient-Reported Concept Description of Bowel Urgency**

| **Patient-reported description of bowel urgency** | | **Frequency of participant reports n (%)** | |
| --- | --- | --- | --- |
| **UC (N=15)** | **CD (N=15)** | **Aspect of bowel urgency experience that is most bothersome^†,‡^** | **Aspect of bowel urgency that is meaningful to improve^†,‡^** |
| Bowel urgency was described by participants with UC as having a sudden and/or strong urge to use the bathroom for a bowel movement, not being able to wait, and having a few minutes to get to the bathroom. Participants with UC described aspects of bowel urgency in the following ways:  **Severity:** Participants described bowel urgency severity as not too severe to very severe, extreme urgency/pain, and increased frequency and duration of urgency. Participants defined bowel urgency severity as the frequency of bowel movements or needing to return to bathroom due to unfinished bowel movements, how urgently or quickly one needs to go to the bathroom, the amount of pain and/or cramping that occurs, the lack of control over urgency, the amount of discharge or diarrhea during a bowel movement, how tired one gets when urgency occurs, the consistency of stool, and whether accidents occur  **Frequency:** Bowel urgency frequency varied greatly between individuals and ranged from multiple times a day to monthly.  **Duration:** Bowel urgency duration varied between individuals, lasting between a couple seconds to the entirety of a day.  **Occurrence:** Participants described experiencing bowel urgency when they were stressed, eating certain food, and/or at certain times of the day (e.g., consistently in the mornings). | Bowel urgency was described by participants with CD as a strong and sudden need to use the bathroom, not being able to wait, having a few minutes to get to the bathroom, feeling as if one has been holding a bowel movement in for hours, or feeling like one has gas, but then passes stool. Participants with CD described aspects of bowel urgency in the following ways:  **Severity:** Participants described severity as the amount of time they have or not being able to wait at all before needing to use the restroom, the frequency or duration of bowel urgency, increased and uncontrolled symptomology associated with bowel urgency (abdominal pain, overall symptoms, irritation, constipation), having a flare-up, dependent upon the severity of resulting bowel movement, and accidents.  **Frequency:** Bowel urgency frequency varied greatly between individuals with some experiencing multiple times a day to a monthly basis.  **Duration:** Bowel urgency duration varied between individuals, lasting between five minutes to all day, or until an individual uses the restroom to relieve themselves.  **Occurrence:** Participants described experiencing bowel urgency at different times of the day, with some participants noting that bowel urgency occurred after meals and some experiencing bowel urgency only during a flare-up. | All aspects of urgency (CD: n=3)  ***Frequency aspect***  Frequency of bowel urgency (UC: n=3)  Having to go to the bathroom urgently or repeatedly first thing in the morning (UC: n=2)  ***Severity aspect***  Urgent need to use the bathroom or need to be near a bathroom (CD: n=2)  ***Other symptoms***  Experiencing abdominal pain or abdominal cramps (UC: n=6)  ***Impact of urgency***  Having accidents as a result of bowel urgency (UC: n=2)  Uncertainty of condition (CD: n=2)  Fear of experiencing accident or soiling clothes (CD: n=2)  Every aspect of bowel urgency/bowel movement (UC: n=1; CD: n=1)  Mental aspect of urgency (CD: n=1)  Feeling the need to stop in every bathroom just in case one needs to have a bowel movement (UC: n=1)  Inconvenience of having to stop what one is doing to find a bathroom because of urgency (UC: n=1)  Lack of control of urgency (UC: n=1)  Needing to be always near a bathroom (UC: n=1)  Needing to plan to be prepared in case of urgency (UC: n=1)  Not being able to finish eating (UC: n=1) | Decreased flares (UC: n=1)  Decreased accidents (UC: n=1)  ***Frequency aspect***  Decreased frequency of bowel urgency (UC: n=5, CD: n=5)  Complete resolution/‌not needing to go to the bathroom as frequently (UC: n=1, CD: n=4)  ***Severity aspect***  Decrease in the severity of bowel urgency (CD: n=1, UC: n=4)  Being able to delay urgency (UC: n=1)  Improved control (UC: n=3)  Having more time to get to the bathroom (CD: n=3)  ***Impact of urgency***  Being able to eat a regular diet (CD: n=1)  Having urgency outside of the home (UC: n=1)  ***Co-occurring symptoms***  Abdominal pain (CD: n=3, UC: n=4)  Diarrhea (CD: n=1)  Abdominal cramping (UC: n=1)  Fatigue (UC: n=1) |

CD, Crohn’s disease; UC, ulcerative colitis

n= frequency of report of concepts/domains

^†^Counts are not mutually exclusive.

^‡^Not all participants were asked what aspect of bowel urgency was most bothersome or meaningful to improve or provided interpretable responses to those probes.

**Supplementary Table 3. Patient-Reported Concept Description of Bowel Urgency-Related Accidents**

| **Patient-reported description of bowel urgency-related accidents** | | **Aspects of bowel urgency-related accidents that are meaningful to improve^†;‡^** |
| --- | --- | --- |
| **UC (N=13)** | **CD (N=15)** |  |
| Bowel urgency-related accidents were described by participants with UC as being unable to make it to the bathroom, stool consistency described as a leak, liquid stool, or bowel movement that can be mistaken as gas or accompanied by bowel urgency. bowel urgency-related accidents were reported to occur more often during a flare-up. Participants described the aspects of bowel urgency-related accidents as follows:  **Frequency:** Participants described bowel urgency-related accidents as occurring one to three times a week, every three weeks, to a few times a year.  **When:** Participants described bowel urgency-related accidents as occurring when one does physical activity; in the mornings, after dinner, after one eats a meal, when one eats certain foods, or at random times during the day; can occur at night.  **Stool during accidents:** Participants described stool during accidents as diarrhea, or a loose, watery, liquid, mush, pudding-like, soft, or runny bowel movement; can sometimes be solid. | Bowel urgency-related accidents were described by participants with CD as having little or no control of bowels, which occurs at home, at work, or when in public; participants describe different scenarios in which their accidents are preceded by a feeling of urgency or occur spontaneously with little to no indication. Participants described the aspects of bowel urgency-related accidents as follows:  **Frequency:** Participants described bowel urgency-related accidents as occurring from two times a day to once every two to six months.  **When:** Participants reported experiencing bowel urgency-related accidents at various times of day or night, with either a pattern of specific timing or no consistent timing at all.  **Stool during accidents:** Participants described stool during accidents as liquid, runny stool, watery/loose stool, stool containing mucus, stool similar to diarrhea, smaller stool, unformed stool in comparison to typical solid stool. | ***Severity aspect***  Lessened urgency (CD n=3)  Less severe accidents (CD n=2)  More time to hold in a bowel movement (CD n=1)  More control over aspects of accidents (CD n=1)  ***Frequency aspect***  No experience of bowel urgency-related accidents at all (UC n=4; CD n=3)  Fewer accidents (CD n=2)  No experience of bowel urgency at all related to bowel urgency-related accidents (UC n=1)  ***Duration aspect***  Flares to resolve in a shorter amount of time (CD n=1) |

CD, Crohn’s disease; UC, ulcerative colitis

n= frequency of report of concepts/domains

^†^Counts are not mutually exclusive

^‡^Not all participants were asked what aspect of bowel urgency-related accidents is meaningful to improve

**Supplementary Table 4.** **Patient-Reported Concept Descriptions of Inflammatory Bowel Disease Signs, Symptoms, and Quality-Of-Life Impacts Experienced with Bowel Urgency**

| **Concepts reported by participants** | **UC** | | **CD** | |
| --- | --- | --- | --- | --- |
|  | **Frequency of participant reports (N=15), n (%)** | **Concept description** | **Frequency of participant reports (N=15), n (%)** | **Concept description** |
| ***IBD signs and symptoms experienced with bowel urgency*** | | | | |
| **Abdominal pain^**,†^** | 13 (86.7%) | A sharp painful feeling in the stomach/intestinal area that occurs with cramping in many instances as a result of UC and/or bowel urgency movements | 13 (86.7%) | A sharp feeling like knives or like getting hit in the gut. Other participants described as a dull pain similar to a stomachache or bellyache. Participants used the terms stomach or belly ache, stomach pain, or abdominal pain to refer to abdominal pain. |
| **Fatigue** | 12 (80.0%) | A feeling of tiredness, weakness, exhaustion, lack of energy, dehydration, and being worn out due to bowel urgency | 13 (86.7%) | Tiredness or lack of energy that often occurs after exerting themselves during bowel movements or caused by stress associated with the experience of bowel urgency |
| **Abdominal cramping^**,†^** | 10 (66.7%) | A churning, twisting, and/or aching feeling that occurs in the stomach area as a result of bowel urgency | 8 (53.3%) | An initial warning signal for a bowel movement, a tightening or restricting of muscles sensation. Some participants described the cramping as a sharp or dull sensation. |
| **Diarrhea** | 5 (33.3%) | Watery, runny, or nonsolid stool occurring with urgency as a result of UC | 4 (26.7%) | Loss of control of bowels occurring as a result of consuming high levels of sugars and coinciding with other symptoms. Two participants did not describe diarrhea in relation to bowel urgency. |
| **Blood in stool** | 4 (26.7%) | Bleeding or having blood in stool when having a bowel movement with urgency or a flare as a result of UC | 4 (26.7%) | Occurring occasionally, with some participants reporting it occurred “sometimes” or “a couple times a year.” Participants did not describe blood in their stool in relation to bowel urgency. |
| **Gas** | 4 (26.7%) | Having and passing gas as a result of bowel urgency | 3 (20.0%) | “Bloating” or an experience where patients believed they were going to have a bowel movement and mostly expelled gas while experiencing bowel urgency |
| **Stomach bubbling/‌rumbling** | 1 (6.7%) | A growling feeling or as if the stomach is talking that occurs with bowel movements during bowel urgency | 4 (26.7%) | Churning, pressure, bubbling, or twisting in a knot feeling while experiencing bowel urgency |
| **Mucus in stool** | 3 (20.0%) | A clear coating or white blobs that coat the stool occurring with bowel urgency as a result of UC | 1 (6.7%) | Experiencing bowel urgency as difficulty determining the contents of their bowel movements due to its liquid consistency |
| **Nausea** | 2 (13.3%) | Feeling sick as a result of urgency | 2 (13.3%) | The feeling of needing to vomit or dry heaving while experiencing bowel urgency |
| **Fever** | 1 (6.7%) | Feeling a hot flush that hits the whole body as a result of bowel urgency and bowel movements that come with nausea | 2 (13.3%) | A part of bowel urgency, with one participant describing chills and sweats |
| **Loss of appetite** | 1 (6.7%) | Described as occurring with bowel urgency | 2 (13.3%) | Described as not wanting to eat whenever experiencing bowel urgency |
| **Constipation** | 1 (6.7%) | Difficulty pushing poop out and sometimes using stool softener as an aid to assist in having a bowel movement | 2 (13.3%) | “Feeling like you have to go but cannot” while experiencing bowel urgency |
| **Headaches** | 0 (0.0%) | Not reported | 2 (13.3%) | Migraines that may have occurred out of nowhere and precede abdominal cramps while experiencing bowel urgency |
| **Sweating** | 2 (13.3%) | Feeling sweaty, occurring with stomach pain, cramping, and urgency | 0 (0.0%) | Not reported |
| **Bloating** | 0 (0.0%) | Not reported | 1 (6.7%) | Feeling bloated while experiencing bowel urgency; no other description was provided |
| **Flushed face** | 1 (6.7%) | Being flushed above the neck that precedes cramping | 0 (0.0%) | Not reported |
| **Rectal itching** | 0 (0.0%) | Not reported | 1 (6.7%) | No additional description was provided |
| **Vomiting** | 0 (0.0%) | Not reported | 1 (6.7%) | Throwing up until there was nothing left, resulting in the need for fluids when hospitalized |
| ***Impacts experienced with bowel urgency*** | | | | |
| **Adaptive behaviors** | **n=15** | | **n=14** | |
| **Dietary changes** | 11 (73.3%) | Only eating certain foods such as cutting out greasy foods, spicy foods, dairy products, and fried foods to mitigate bowel urgency and other symptoms | 13 (86.7%) | Eating smaller amounts of food or eating less frequently, as well as specifically avoiding certain foods like dairy, alcohol, tomatoes, spicy foods, fried foods, and fatty foods in favor of healthier choices to avoid triggering bowel urgency |
| **Bathroom mapping** | 8 (53.3%) | Needing to know where bathrooms are anywhere one goes | 8 (53.3%) | The need to be aware of bathroom locations and as being aware of positioning themselves near bathrooms in public places in order to respond to bowel urgency and avoid any bowel urgency-related accidents |
| **Use of protective undergarments** | 5 (33.3%) | Actively wearing adult diapers or panty liners or having access to them in preparation for bowel urgency or bowel urgency-related accident | 3 (20.0%) | Actively wearing or having access to pads or adult diapers to be prepared for bowel urgency or bowel urgency-related accidents |
| **Ensuring adequate supplies/extra change of clothes** | 0 (0.0%) | Not reported | 3 (20.0%) | Participants making sure they have enough toilet paper, wipes, or other supplies, including their treatment injection to help manage bowel urgency |
| **Meditating** | 0 (0.0%) | Not reported | 2 (13.3%) | No additional description was provided |
| **Use of a heating pad** | 0 (0.0%) | Not reported | 1 (6.7%) | Using a heating pad to help provide symptom relief. Participant did not describe use of a heating pad in relation to bowel urgency |
| **Emotional functioning** | **n=13** | | **n=12** | |
| **Stress** | 6 (40.0%) | Feeling stressed due to bowel urgency pains and needing to know if there is a restroom available or nearby when bowel urgency occurs | 6 (40.0%) | Felt due to the possibility of having an urgency-related accident due to bowel urgency, or the accessibility of a restroom due to bowel urgency |
| **Fear** | 5 (33.3%) | Being concerned about being unable to find a restroom in time when experiencing bowel urgency or afraid of forgetting diapers when traveling | 3 (20.0%) | Being felt at the prospect of having an urgency-related accident due to bowel urgency or experiencing fear due to the level of pain experienced during bowel urgency |
| **Embarrassment** | 5 (26.7%) | Being embarrassed due to bowel urgency- bowel urgency-related accidents and dealing with bowel urgency (e.g., having bowel urgency at a friend’s or while being out, having to wear adult diapers, and the smell from bowel urgency) | 3 (20.0%) | Being felt at having bowel urgency- bowel urgency-related accidents due to bowel urgency or experiencing bowel urgency at a friend’s home or public place |
| **Sadness** | 4 (26.7%) | Feeling down, having dampened spirits, feeling depressed, or feeling bummed out due to the inability to partake in activities such as going out to dinner or hiking due to bowel urgency | 3 (20.0%) | Being felt due to bowel urgency preventing them from doing the things they want to do |
| **Frustration** | 3 (20.0%) | Being upset having to deal with bowel urgency and having to always plan, have accidents, and dealing with the emotional side of the disease | 1 (6.7%) | No additional description was provided |
| **Worry** | 2 (13.3%) | Being concerned about bowel urgency- bowel urgency-related accidents and having to be close to restrooms | 1 (6.7%) | Feeling worried that they might have bowel urgency where there is not a bathroom, that bowel urgency will disrupt their daily activities, and that their bowel urgency could become worse |
| **Decreased mood** | 0 (0.0%) | Not reported | 1 (3.3%) | No additional description was provided |
| **Feeling irritable** | 1 (6.7%) | Not wanting to be around anyone due to dealing with UC | 0 (0.0%) | Not reported |
| **Social functioning** | **n=10** | | **n=13** | |
| **Inability to participate in social activities** | 10 (66.7%) | Not being able to participate or avoiding participating in activities with friends and family in social settings due to bowel urgency | 13 (86.7%) | Having to cancel plans with friends, not attending planned social activities, and being unable to hang out with friends at someone’s house or in public due to bowel urgency |
| **Social isolation** | 1 (6.7%) | Feeling lonely due to dealing with UC | 1 (6.7%) | Staying at home rather than going out due to bowel urgency |
| **Disappointing friends** | 1 (6.7%) | Feeling one’s friends are upset with them for not participating in social activities due to their UC symptoms and flares | 0 (0.0%) | Not reported |
| **Household chores** | **n=9** | | **n=13** | |
| **Difficulty running errands** | 9 (60.0%) | Either delaying, avoiding, or having others run errands due to experiencing bowel urgency and bowel urgency- bowel urgency-related symptoms | 7 (46.7%) | Either delaying, avoiding, or having others run errands due to experiencing bowel urgency and bowel urgency-related symptoms |
| **Inability to do household chores** | 0 (0.0%) | Not reported | 5 (33.3%) | Inability to do household chores, such as cleaning up, due to bowel urgency or bowel urgency-related symptoms such as fatigue, or stomach symptoms |
| **Need to cancel appointments** | 0 (0.0%) | Not reported | 2 (13.3%) | Needing to cancel appointments with a doctor or other appointments due to bowel urgency |
| **Recreational/leisure activities** | **n=6** | | **n=12** | |
| **Difficulty engaging in hobbies or leisure activities** | 3 (20.0%) | Not participating in activities outside of home (e.g., sports, hiking, going to events, or going out to eat) due to UC symptoms | 7 (46.7%) | The inability or impaired ability to shop, paint, go to live music events, bowl, golf, or go on walks due to bowel urgency or bowel urgency-related symptoms |
| **Difficulty exercising** | 3 (20.0%) | Not participating in more strenuous activities (e.g., anything to get the heart moving, sports, hunting, bike riding, walking, and going to the gym) due to UC symptoms | 7 (46.7%) | Difficulty exercising as either avoiding exercise, exercising less intensely, avoiding jogging due to fear of an urgency-related accident, or needing to stop exercising due to bowel urgency or bowel urgency-related symptoms |
| **Sleep** | **n=8** | | **n=10** | |
| **Interrupted sleep** | 8 (53.3%) | Not being able to sleep through the night without waking up due to UC symptoms | 10 (66.7%) | Being woken up in the middle of the night due to bowel urgency and needing to use the bathroom |
| **Travel** | **n=9** | | **n=9** | |
| **Difficulty commuting** | 8 (53.3%) | Having trouble with daily activities that required traveling due to bowel urgency (e.g., having to be in the car for more than a short car ride, having bowel urgency while in the car, and/or not having a place to stop to use the bathroom while on the road) | 0 (0.0%) | Not reported |
| **Inability to travel** | 0 (0.0%) | Not reported | 5 (33.3%) | Being unable to or having difficulty with daily travel and vacation travel. Daily travel was described as being unable to or having difficulty driving long distances, using transportation such as trains, due to bowel urgency and bowel urgency-related symptoms. Vacation travel was described as being unable to or having difficulty engaging in other forms of vacation travel, such as flying, due to bowel urgency and bowel urgency-related symptoms. |
| **Need to delay travel** | 0 (0.0%) | Not reported | 4 (26.7%) | Either stopping their daily traveling order to use the restroom, being prevented from beginning travel due to the need to use the restroom or needing to return home in the middle of their trip to use the restroom due to bowel urgency |
| **Avoid daily travel** | 2 (13.3%) | Having trouble with going anywhere for day-to-day activities that required long or short distance traveling due to bowel urgency | 0 (0.0%) | Not reported |
| **Travel precautions** | 0 (0.0%) | Not reported | 2 (13.3%) | Eating less, watching their diet, and making sure they have the necessary medications prior to traveling on vacation due to bowel urgency |
| **Impacted vacation travel** | 1 (6.7%) | Having difficulty with going on trips especially when UC is flaring | 0 (0.0%) | Not reported |
| **Work** | **n=7** | | **n=10** | |
| **Interrupted work activities** | 0 (0.0%) | Not reported | 6 (40.0%) | Described as bowel urgency interrupting their work, such as frequent bathroom breaks |
| **Impaired ability to work** | 5 (33.3%) | Having difficulty with finding a job that is suitable, going to work, and doing their on-the-job tasks | 0 (0.0%) | Not reported |
| **Need to miss work** | 0 (0.0%) | Not reported | 5 (33.3%) | Needing to miss work due to bowel urgency such as needing to use the restroom prior to going to work, and bowel urgency-related symptoms, such as pain and fatigue, or experiencing bowel urgency and bowel urgency-related accidents |
| **Extensive time in bathroom at work** | 2 (13.3%) | Having to be on the toilet for long periods of time or keep going to the bathroom while working due to UC symptoms | 0 (0.0%) | Not reported |
| **Family activities** | **n=4** | | **n=7** | |
| **Inability to participate in family activities** | 4 (26.7%) | Not being able to join in on family activities (e.g., playing cards, visiting, or hanging out with family) due to bowel urgency | 7 (46.7%) | Being unable to go to relatives’ homes, being unable to go shopping with family, being unable to spend more time with grandchildren, leaving the dinner table, and being unable to do activities with their significant other due to bowel urgency and bowel urgency-related symptoms |
| **Sexual health** | **n=5** | | **n=5** | |
| **Impacted sexual activity** | 5 (33.3%) | Unable to engage in or feeling awkward or hesitant to engage in sex-related activities due to bowel urgency | 5 (33.3%) | Avoiding or decreased sexual activity as actively avoiding or not wanting to engage in sexual activity due to bowel urgency, bowel urgency-related symptoms, such as pain, and flares |
| **Independence** | **n=0** | | **n=6** | |
| **Dependence on others** | 0 (0.0%) | Not reported | 4 (26.7%) | Needing to ask others to go to the store and needing assistance from others to do tasks due to bowel urgency or bowel urgency-related symptoms |
| **Inability to go out alone** | 0 (0.0%) | Not reported | 2 (13.3%) | Not going anywhere independently due to bowel urgency, bowel urgency-related symptoms, or in the event of a bowel urgency-related accident |
| **Self-image** | **n=3** | | **n=3** | |
| **Low self-esteem** | 3 (20.0%) | Not feeling positive about oneself due to UC symptoms, and impacts of medication for UC | 2 (13.3%) | Feeling self-conscious with friends or significant others due to bowel urgency |
| **Decreased confidence** | 0 (0.0%) | Not reported | 1 (6.7%) | Not having confidence in oneself due to bowel urgency |
| **Self-pity** | 1 (6.7%) | Feeling sorry for oneself and disappointed when thinking about UC | 0 (0.0%) | Not reported |
| **Caregiver responsibilities** | **n=2** | | **n=3** | |
| **Inability to perform caregiver responsibilities** | 1 (6.7%) | Not being able to care for child due to UC symptoms | 3 (20.0%) | Needing to leave children’s school functions or pick grandchildren up from school, extracurricular activities, sporting events, and being unable to take care of others due to bowel urgency and fatigue from bowel urgency |
| **Difficulty helping family** | 1 (6.7%) | Unable ability to care for family a quickly as possible due to UC symptoms being an inconvenience | 0 (0.0%) | Not reported |
| **Cognitive functioning** | **n=2** | | **n=2** | |
| **Inability to concentrate** | 2 (13.3%) | Concentration being impeded due to the nature of UC symptoms and frequency/duration of bowel movements | 2 (13.3%) | Difficulty focusing on necessary tasks, meetings, or social activities due to bowel urgency and lack of energy from bowel urgency |
| **Activities of daily living** | **n=3** | | **n=0** | |
| **Interrupted eating** | 3 (20.0%) | Needing to use the bathroom while eating a meal or waiting to eat until later in the day to avoid UC symptoms | 0 (0.0%) | Not reported |
| **Spouse/partner relationships** | **n=1** | | **n=1** | |
| **Difficulty communicating with significant other** | 0 (0.0%) | Not reported | 1 (6.7%) | No additional description was provided |
| **Difficulty explaining disease** | 1 (6.7%) | Unable to easily define UC to others, especially in intimate relationships | 0 (0.0%) | Not reported |
| **Physical functioning** | **n=1** | | **n=0** | |
| **Impaired walking** | 1 (6.7%) | Difficulty moving around on foot due to their bowel urgency | 0 (0.0%) | Not reported |
| **School** | **n=0** | | **n=1** | |
| **Missing school** | 0 (0.0%) | Not reported | 1 (6.7%) | Missing classes or tests during schools due to their experience of bowel urgency |

Note: The table presents the sign and symptom concepts in order of frequency of total sample report (i.e., most to least frequent); impacts are presented in order of most frequently reported domain in the total sample, then impact concepts within each domain are presented in order of frequency of total sample report. Percentages are calculated based on the total number of participants in each group (UC: N=15, CD: N=15)

^**^UC: Four participants reported abdominal pain and abdominal cramping to be the same; ten reported abdominal pain and abdominal cramping to be different

^†^CD: Four participants reported abdominal cramping and abdominal pain to be the same; six reported abdominal pain and abdominal cramping to be different

CD, Crohn’s disease; IBD, inflammatory bowel disease; UC, ulcerative colitis

N= Total number of participants in each group

n=Frequency of report of concepts/domains

**Supplementary Table 5. Patient-Reported Concept Descriptions of Inflammatory Bowel Disease Signs, Symptoms, and Quality-Of-Life Impacts Experienced with Bowel Urgency-Related Accidents**

| **Concept reported by participants** | **UC** | | **CD** | |
| --- | --- | --- | --- | --- |
|  | **Frequency of participant reports (N=13), n (%)** | **Concept description** | **Frequency of participant reports (N=15), n (%)** | **Concept description** |
| ***IBD signs and symptoms experienced with bowel urgency-related accidents*** | | | | |
| **Abdominal pain** | 10 (76.9%) | Stomach hurting, “bubble guts,” upset stomach or stomachache, or a sharp, dull, or uncomfortable pain | 10 (66.7%) | Stomach rumbling, belly or stomach aches, and sharp pains, noting that their pain typically occurred before, during, and after accident |
| **Abdominal cramping** | 9 (69.2%) | Feeling like you have to pass gas or period cramping; sometimes an indication to find a bathroom; more intense than non-accident cramping sensation | 9 (60.0%) | Described as the build up to an accident, stomach rumbling, discomfort or as a watery/bubbly feeling in the stomach, noting that their cramps typically occurred consistently or before, during, and after accidents |
| **Fatigue** | 7 (53.8%) | Feeling “exhausted,” “like a lot has been taken out of you,” or feeling tired following an accident | 10 (66.7%) | A feeling of tiredness or lethargy that co-exists with other symptoms of CD occurring both before and after accidents |
| **Gas** | 10 (76.9%) | Experiencing gas during an urgency-related accident | 7 (46.7%) | An event that precedes or occurs during accidents that may indicate that the participant has had an accident; participants describe flatulence as gas that happens during a leak/accident that includes feelings like bloating and pressure |
| **Mucus in stool** | 8 (61.5%) | A clear, white, filmy, or liquid mucus with a bowel movement during an accident | 8 (53.3%) | Various quantities of greasy, oily, yellowish-looking substance that is visible in the unformed stool and occurs during an accident. |
| **Loss of appetite** | 5 (38.5%) | Not wanting to eat anything while experiencing accidents or after experiencing an accident | 8 (53.3%) | A lack of desire and/or motivation to eat, leading to weight fluctuation, diarrhea, and a fear of vomiting up food. Participants noted loss of appetite preceded or followed accidents. |
| **Blood in stool** | 5 (38.5%) | Seeing visible blood in the stool during an accident that occurs every now and then to always for some individuals | 5 (33.3%) | The appearance of various quantities of blood, ranging from occasional to minimal blood that appears in their stool |
| **Stomach rumbling/‌bubbling** | 0 (0.0%) | Not reported | 3 (20.0%) | A stomach-churning sensation when bowel accidents due to CD occur; participants note that stomach rumbling varies between occurring sometimes and always |
| **Constipation** | 0 (0.0%) | Not reported | 1 (6.7%) | A lack of ability to produce solid, non-watery stool even if they were experiencing bowel urgency |
| **Nausea** | 0 (0.0%) | Not reported | 1 (6.7%) | Occurring during accidents, in conjunction with abdominal pain and/or cramping; participants also reported experiencing feeling the need to vomit, but no vomit is expelled in addition to dry heaving |
| **Rectal itching** | 0 (0.0%) | Not reported | 1 (6.7%) | No additional description was provided |
| **Vomiting** | 0 (0.0%) | Not reported | 1 (6.7%) | Throwing up when one is having an accident |
| ***Impacts experienced with bowel urgency-related accidents*** | | | | |
| **Adaptive behaviors** | **n=13** | | **n=15** | |
| **Dietary changes** | 5 (38.5%) | Avoiding certain foods and drinks such as milk, cheese, wheat bread, creams or sauces, or alcohol; not being able to enjoy foods one used to | 12 (80.0%) | Avoiding certain foods/food groups, including wine, dairy, spicy food, junk food, coffee, alcohol, fried foods, steak, and tomatoes to mediate the effects of bowel accidents due to CD |
| **Bathroom mapping** | 8 (61.5%) | Needing to know where bathrooms are and if bathrooms are available when going out in order to avoid having an accident | 6 (40.0%) | Having to constantly be aware of restroom locations to mediate the effects of bowel accidents due to CD, noting the impact it has on their daily travel and the feeling of safety they experience with the knowledge they are close to a restroom |
| **Use of protective undergarments** | 5 (38.5%) | Putting toilet paper in underwear or wearing diapers, such as Depends to avoid accidents on clothes | 5 (33.3%) | Wearing protective undergarments, such as diapers, shields, or pads, and bringing them with them to mediate the effects of bowel accidents due to CD |
| **Ensuring adequate supplies/ extra change of clothes** | 5 (38.5%) | Carrying an extra set of clothes to change into in case of an accident | 6 (40.0%) | Always being prepared with extra clothes/underwear to mediate the effects of bowel accidents due to CD. Supplies are described as an extra pair of clothes/underwear, toilet paper, baby wipes, “itchy medication,” and a diaper bag. Participants also describe changing clothes to mediate the effects of bowel accidents due to CD. |
| **Change of daily routine** | 2 (15.4%) | Having to use the bathroom in the mornings or before going out to avoid any accidents | 0 (0.0%) | Not reported |
| **Meditating** | 0 (0.0%) | Not reported | 2 (13.3%) | Using meditation and yoga to mediate the stress and other effects of bowel accidents due to CD |
| **Carrying injectable medication** | 0 (0.0%) | Not reported | 1 (6.7%) | Carrying ready for use injectable medication to mediate the effects of bowel accidents due to CD; noting they use it more during travel |
| **Emotional functioning** | **n=10** | | **n=13** | |
| **Embarrassment** | 8 (61.5%) | Not wanting to have an accident in public or around others; emotional to deal with or have others deal with it | 9 (60.0%) | A feeling that comes with having an accident outside of one’s own home and/or in the company of others |
| **Fear** | 3 (23.1%) | Felt when having an accident while traveling or preparing for an accident | 4 (26.7%) | Constantly being afraid, fearful, and scared of having an unexpected accident when traveling, spending time with family, and engaging in other social activities |
| **Stress** | 2 (15.4%) | Felt in response to needing to know where the bathrooms are located | 5 (33.3%) | Anxiety due to fear of having an accident and the idea that no one else knows what is going on, noting they mediate the stress using jokes |
| **Worry** | 4 (30.8%) | Feeling nervous, panicky, or worried that one will have an accident | 1 (6.7%) | Feeling afraid their CD will consistently get worse throughout their day |
| **Sadness** | 2 (15.4%) | Feeling bad, down, or sad due to potential accidents | 2 (13.3%) | Feeling upset that is associated with low self-esteem, not feeling like oneself, and lack of control over one’s life |
| **Anger** | 1 (7.7%) | Feeling angry due to accidents | 2 (13.3%) | Feeling mad to the point of hitting furniture, stemming from pain, and lack of control over one’s own body |
| **Devastation** | 2 (15.4%) | Feeling despair or devastation due to accidents | 0 (0.0%) | Not reported |
| **Frustration** | 0 (0.0%) | Not reported | 2 (13.3%) | Feeling disappointed in oneself if one were to have an accident |
| **Negatively impacted mood** | 0 (0.0%) | Not reported | 2 (13.3%) | Being stressed, tired, and feeling down due to bowel accidents due to CD, attributing being not in the best of moods to stress and fatigue |
| **Disgust** | 1 (7.7%) | Feeling disgust due to accidents | 0 (0.0%) | Not reported |
| **Feeling lonely** | 1 (7.7%) | Felt in response to self-isolation due to the possibility of an urgency-related accident | 0 (0.0%) | Not reported |
| **Social functioning** | **n=8** | | **n=10** | |
| **Inability to participate in social activities** | 8 (61.5%) | Inability to participate or avoiding participating in activities with friends and family in social settings due to bowel urgency | 4 (26.7%) | Unable to participate in social activities such as drinking alcohol, seeing grandchildren, seeing live shows or going out with friends due to bowel accidents due to CD |
| **Social isolation** | 0 (0.0%) | Not reported | 8 (53.3%) | Feeling left out and/or exasperated, needing to stay home, feelings of embarrassment, fear of having an accident, and feeling as though they are prevented from being outgoing |
| **Changing plans** | 0 (0.0%) | Not reported | 2 (13.3%) | Done in order to avoid having an accident in front of others or leaving events early to mediate the effects of bowel accidents due to CD |
| **Travel** | **n=7** | | **n=9** | |
| **Avoid or delay travel** | 2 (15.4%) | Done in case of needing a bathroom urgently to avoid a potential accident outside of the home | 4 (26.7%) | Not traveling due to bowel accidents due to CD and the practices they take when they do travel, such as staying close to a restroom or only traveling short distances |
| **Inability to travel** | 0 (0.0%) | Not reported | 4 (26.7%) | Not being able to take vacations or different methods of travel (e.g., trains) because of unpredictable accidents due to CD |
| **Unable to do routine travel** | 4 (30.8%) | Being unable to drive for longer than 15 miles or use public transportation to avoid accidents | 0 (0.0%) | Not reported |
| **Difficulty traveling for vacation** | 2 (15.4%) | Difficulty traveling on airplanes due to potential accidents | 0 (0.0%) | Not reported |
| **Difficulty preparing for travel** | 1 (7.7%) | Time spent on preparing for travel such as preparing diapers and extra clothes | 0 (0.0%) | Not reported |
| **Interrupted by urgency during travel** | 0 (0.0%) | Not reported | 1 (6.7%) | Being interrupted by the potential of an accident, noting the inconvenience of turning their car around on their way to a destination |
| **Household chores** | **n=4** | | **n=10** | |
| **Inability to run errands** | 0 (0.0%) | Not reported | 7 (46.7%) | Unable to go to the doctor, the pharmacy, the store, or perform other errands; noting that they would not go out if they were experiencing a flare-up or a “bad day” and the modifications they make (e.g., ordering online) |
| **Avoid running errands** | 4 (30.8%) | Not running errands or leaving the house to avoid accidents | 0 (0.0%) | Not reported |
| **Inability to do household chores** | 0 (0.0%) | Not reported | 4 (26.7%) | Lack of ability to clean the house or go shopping, citing reasons such as fatigue, effects of strenuous work, and fear of a flare-up |
| **Need to cancel appointments** | 0 (0.0%) | Not reported | 1 (6.7%) | Cancelling business and doctor’s appointments, among other appointments, due to bowel accidents due to CD |
| **Recreational/leisure** | **n=4** | | **n=10** | |
| **Difficulty exercising** | 2 (15.4%) | Exercising less or not at all to avoid accidents | 6 (40.0%) | Not being able to perform activities that they previously were able to engage in, such as running, walking, and activities requiring endurance |
| **Difficulty participating in hobbies** | 2 (15.4%) | Difficulty concentrating when watching TV, reading, unable to do outdoor hobbies, such as working on one’s care due to inability to squat and the associated risk of leaks due to accidents | 2 (13.3%) | Not participating in activities that were strenuous and/or took place outside or somewhere were a restroom is not accessible |
| **Inability to do outdoor activities** | 0 (0.0%) | Not reported | 3 (20.0%) | Inability to walk outside or walk somewhere without access to a restroom, due to bowel accidents due to CD |
| **Independence** | **n=1** | | **n=7** | |
| **Dependence on others** | 0 (0.0%) | Not reported | 2 (13.3%) | Restricting the participants from doing things they want to do, noting they cannot care for others as they desire |
| **Inability to go out alone** | 0 (0.0%) | Not reported | 2 (13.3%) | Not feeling safe to leave home in case they have an accident away from home without someone to assist them |
| **Requiring assistance** | 0 (0.0%) | Not reported | 2 (13.3%) | Needing help with tasks such as going to the store or pharmacy, due to bowel accidents due to CD |
| **Lack of control over daily life** | 0 (0.0%) | Not reported | 1 (6.7%) | Lack of control over daily life due to bowel accidents due to CD as feeling controlled by their experiences with CD |
| **Need others to run errands** | 1 (7.7%) | Having others run errands to avoid leaving the house | 0 (0.0%) | Not reported |
| **Work** | **n=2** | | **n=6** | |
| **Interrupted work activities** | 0 (0.0%) | Not reported | 6 (40.0%) | Experiencing a lack of control over accidents that impacts daily work tasks, work meetings, and work events. Participants describe their experience of jobs not allowing employees to continuously go to the restroom. Participants note that working from home has proven to be a suitable alterative. |
| **Decreased productivity** | 1 (7.7%) | Having to leave work or being less productive at work due to accidents | 0 (0.0%) | Not reported |
| **Have to leave work** | 1 (7.7%) | Having to leave work due to accidents | 0 (0.0%) | Not reported |
| **Taking days off** | 0 (0.0%) | Not reported | 1 (6.7%) | Needing to stay home due to fatigue and bowel urgency |
| **Sleep** | **n=2** | | **n=5** | |
| **Interrupted sleep** | 2 (15.4%) | Being woken up during the night due to or to avoid accidents | 5 (33.3%) | Shortened and interrupted sleep, nighttime awakenings, pain, urges that arise while sleeping, and accidents while sleeping |
| **Physical functioning** | **n=2** | | **n=3** | |
| **Unable to perform physical activities** | 2 (15.4%) | Inability to perform physical activities such as squatting due to risk of leaks and walking due to discomfort | 2 (13.3%) | Lack of ability to run, work out strenuously, and take walks with their partner |
| **Decreased endurance** | 0 (0.0%) | Not reported | 1 (6.7%) | Inability to perform exercise for a lengthy period of time |
| **Self-image** | **n=1** | | **n=4** | |
| **Low self-esteem** | 1 (7.7%) | Feeling different and not normal | 3 (20.0%) | Feelings of embarrassment, and self-consciousness |
| **Decreased confidence** | 0 (0.0%) | Not reported | 1 (6.7%) | Lack of confidence in comparison to the confidence they had before experiencing accidents due to CD |
| **Self-disgust** | 0 (0.0%) | Not reported | 1 (6.7%) | The rough emotional feeling that comes after an accident, noting that it affected the urgency that they respond to future signs of an incoming accident |
| **Sexual health** | **n=0** | | **n=5** | |
| **Avoiding or decreased sexual activity** | 0 (0.0%) | Not reported | 5 (33.3%) | Avoiding or decreasing sexual activity was described as not engaging in sexual contact due to pain or flare-ups and as sleeping in a different bed than their partner, as well as a lower level of sexual activity |
| **Family activities** | **n=0** | | **n=3** | |
| **Inability to participate in family activities** | 0 (0.0%) | Not reported | 3 (20.0%) | Not being able to participate in activities due to bowel accidents due to CD, giving examples such as family events and social gatherings, because of flare-ups or not having access to a restroom |
| **Spouse/partner relationships** | **n=1** | | **n=2** | |
| **Difficulty communicating with significant other** | 1 (7.7%) | Difficulty in having to explain the condition to a potential partner | 1 (6.7%) | The concept of being open with one’s partner around restrictions on activities as being more difficult |
| **Limited activities with spouse** | 0 (0.0%) | Not reported | 1 (6.7%) | Limiting or “cutting back” on activities that were enjoyable for participants to engage in with their spouse |
| **Caregiver responsibilities** | **n=1** | | **n=1** | |
| **Unable to perform caregiver responsibilities** | 1 (7.7%) | Being unable to care for family, such as preparing dinner due to accidents | 1 (6.7%)) | Inability to drop grandchildren off at school due to flare-up of bowel accidents that require them to need back up plans |
| **School** | **n=0** | | **n=1** | |
| **Missing school** | 0 (0.0%) | Not reported | 1 (6.7%) | Missing classes and tests, noting that this can be mediated by open communication with educators |

Note: The table presents the sign and symptom concepts in order of frequency of total sample report (i.e., most to least frequent); impacts are presented in order of most frequently reported domain in the total sample, then impact concepts within each domain are presented in order of frequency of total sample report. Percentages are calculated based on the total number of participants in each group (UC: N=13, CD: N=15)

CD, Crohn’s disease; IBD, inflammatory bowel disease; UC, ulcerative colitis

N= Total number of participants in each group

n=Frequency of report of concepts/domains

**Supplementary Table 6. Saturation Grid for the Crohn’s Disease Group**

| Root concept or domain (n) | Participants with CD (N=15) | | | |
| --- | --- | --- | --- | --- |
|  | Transcript group 1^†^ (n=4) | Transcript group 2^‡^ (n=4) | Transcript group 3^§^ (n=4) | Transcript group 4^**^ (n=3) |
| Bowel urgency | | | | |
| Sign and symptom concepts occurring with the experience of bowel urgency | | | | |
| Abdominal pain (n=11) | X |  |  |  |
| Fatigue (n=8) | X |  |  |  |
| Abdominal cramping (n=5) | X |  |  |  |
| Blood in stool (n=4) | X |  |  |  |
| Diarrhea (n=4) | X |  |  |  |
| Stomach rumbling/bubbling (n=4) | X |  |  |  |
| Gas (n=3) | X |  |  |  |
| Constipation (n=2) | X |  |  |  |
| Fever (n=2) | X |  |  |  |
| Headaches (n=2) |  |  | X |  |
| Nausea (n=2) |  | X |  |  |
| Loss of appetite (n=1) | X |  |  |  |
| Bloating (n=1) | X |  |  |  |
| Rectal itching (n=1) | X |  |  |  |
| Vomiting (n=1) |  | X |  |  |
| Domains of impacts associated with the experience of bowel urgency | | | | |
| Adaptive behaviors (n=14) | X |  |  |  |
| Household chores (n=13) | X |  |  |  |
| Social functioning (n=13) | X |  |  |  |
| Emotional functioning (n=12) | X |  |  |  |
| Recreational/leisure activities (n=12) | X |  |  |  |
| Sleep (n=10) | X |  |  |  |
| Work (n=10) | X |  |  |  |
| Travel (n=9) | X |  |  |  |
| Family activities (n=7) | X |  |  |  |
| Independence (n=6) | X |  |  |  |
| Sexual health (n=5) | X |  |  |  |
| Caregiver responsibilities (n=3) |  | X |  |  |
| Self-image (n=3) |  | X |  |  |
| Cognitive functioning (n=2) | X |  |  |  |
| School (n=1) |  | X |  |  |
| Spouse/partner relationships (n=1) |  |  |  | X |
| Bowel urgency-related accidents | | | | |
| Sign and symptom concepts occurring with the experience of bowel urgency-related accidents | | | | |
| Abdominal pain (n=9) | X |  |  |  |
| Fatigue (n=7) | X |  |  |  |
| Abdominal cramping (n=5) | X |  |  |  |
| Gas (n=3) | X |  |  |  |
| Stomach rumbling/bubbling (n=3) | X |  |  |  |
| Blood in stool (n=2) | X |  |  |  |
| Loss of appetite (n=2) | X |  |  |  |
| Constipation (n=1) |  |  | X |  |
| Nausea (n=1) |  | X |  |  |
| Rectal itching (n=1) | X |  |  |  |
| Vomiting (n=1) |  | X |  |  |
| Domains of impacts associated with the experience of bowel urgency-related accidents | | | | |
| Adaptive behaviors (n=15) | X |  |  |  |
| Emotional functioning (n=13) | X |  |  |  |
| Household chores (n=10) | X |  |  |  |
| Recreational/leisure activities (n=10) | X |  |  |  |
| Social functioning (n=10) | X |  |  |  |
| Travel (n=8) | X |  |  |  |
| Independence (n=7) | X |  |  |  |
| Work (n=6) | X |  |  |  |
| Sexual health (n=5) | X |  |  |  |
| Sleep (n=5) |  | X |  |  |
| Self-image (n=4) |  | X |  |  |
| Family activities (n=3) | X |  |  |  |
| Physical functioning (n=3) | X |  |  |  |
| Spouse/partner relationships (n=2) |  |  |  | X |
| Caregiver responsibilities (n=1) |  | X |  |  |
| School (n=1) |  | X |  |  |

N=Total number of participants in each group

n=Number of spontaneous sign/symptom reports

CD, Crohn’s disease

^†^Participants included in transcript Group 1: 20-02, 40-01, 40-02, 40-04

^‡^Participants included in transcript Group 2: 20-03, 20-04, 30-04, 40-07

^§^Participants included in transcript Group 3: 40-08, 90-01, 90-03, 90-04

^**^Participants included in transcript Group 4: 40-09, 40–10, 40-11
